# Extracorporeal membrane oxygenation in adult patients with congenital heart disease

**DOI:** 10.1007/s12471-014-0526-z

**Published:** 2014-02-13

**Authors:** R. J. Uilkema, L. C. Otterspoor

**Affiliations:** 1 Division Vital Functions, Intensive Care Centre, University Medical Centre, Utrecht, the Netherlands; 2 Present Address: Department of Cardiology, Catharina Hospital, Eindhoven, the Netherlands

## Background

Due to improved diagnostic and therapeutic methods, the prognosis of patients with congenital heart disease has improved dramatically [1, 2]. Therefore, a considerable number of these patients survives to adulthood. As a consequence, an increased number of them will eventually develop complications, varying from mild rhythm disturbances to decompensated heart failure or cardiogenic shock. Furthermore, because of improved survival of patients with congenital heart disease, more re-operations are necessary [3].

**Fig. 1:**
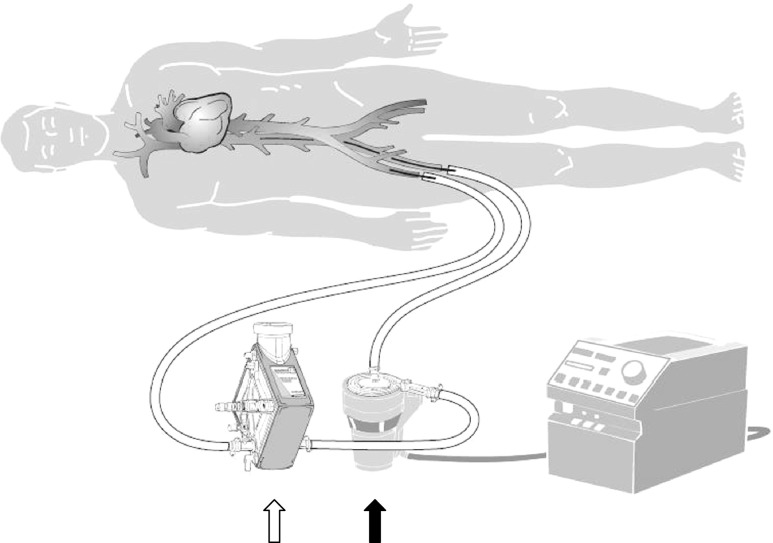
Schematic drawing of a veno-arterial ECMO system. Blood is pumped from a large vein to an artery. The system contains a pump (*black arrow*) and an oxygenator (*white arrow*). A heater is needed to prevent the extracorporeal blood from cooling off

Extracorporeal membrane oxygenation (ECMO) is a support system that consists of a pump and an oxygenator (Fig. 1). Blood is pumped from a large vein through an oxygenator, and back into an artery. Therefore, the system functions as a complete cardiac and pulmonary serial bypass. The cannulas can be inserted into either a central or peripheral vein or artery (Fig. 1)

In acute cardiogenic shock, ECMO can be used to provide circulatory and respiratory support and serves as a bridge-to-decision.

Furthermore, because of improved survival to adulthood of patients with congenital heart disease, more re-operations are necessary [3]. ECMO can be used as bridge-to-surgery, but also to provide circulatory and respiratory support in postcardiotomy cardiogenic shock. ECMO can particularly be useful in this patient group who often have a diminished right ventricular function or even a *Fontan* circulation, given that the right ventricle can be fully bypassed.

In this article we describe the use of ECMO in two adult patients with congenital heart disease and shock. The available literature on the use of ECMO in this particular patient group is reviewed.

## Case report 1

A 33-year-old female was admitted to our hospital with acute right-sided heart failure caused by severe outflow tract stenosis. As a child she underwent a correction of a tetralogy of Fallot with closure of the ventricular septum defect and correction of the right ventricular outflow tract. Other medical history revealed bleeding disorders, partially explained by factor V Leiden and hepato-splenomegaly. She rapidly developed severe shock for which a peripheral ECMO was placed (Fig. 1). This stabilised her circulation and allowed careful planning of valve surgery.

Shortly after ECMO implantation, leakage and haematoma at the arterial cannula ensued. This led to a low flow state and subsequent multi-organ failure, including acute-on-chronic kidney failure requiring dialysis. The next day, balloon dilatation of the pulmonary tract and pulmonary valve replacement were performed. Due to new-onset left ventricular failure, probably due to stunning, the patient remained dependent on ECMO.

Six days after correction of the pulmonary stenosis, the ECMO system could be removed. She was almost ready for transfer to the ward when arterial bleeding occurred at the old insertion site of the femoral cannula. Transfusion of blood products and a surgical correction could not prevent her from dying of haemorrhagic shock.

## Case report 2

A 38-year-old male with a Fontan circulation after previous correction of a congenital tricuspid atresia, suffered from recurrent atrial tachyarrhythmias. He was admitted to our hospital for a MAZE operation and partial resection of the atria. Due to an increase in intrathoracic pressure caused by mechanical ventilation, the passive pulmonary blood flow of the Fontan circuit was postoperatively impaired, after which he became hypoxic and hypotensive. In order to optimise pulmonary blood flow, treatment with sedatives, inodilators and nitric oxide ventilation was initiated. Progressive shock and multi-organ failure needing dialysis developed. Eight days after ICU admission an ECMO support system was inserted. After installing ECMO, his haemodynamic condition stabilised, although he was still in need of high-dose vasopressins and fluids. Due to progressive multi-organ failure it was decided to withdraw the ECMO after which he died.

## Discussion

In this case report the use of extracorporeal membrane oxygenation in two patients with congenital heart disease and shock is described. In the first patient, ECMO was primarily used to optimise the clinical condition before operation. This strategy has been used previously, mainly in patients awaiting cardiac or lung transplantation, but also in children awaiting surgery for congenital heart disease [4]. In this case, ECMO was needed to overcome heart failure, one of the major problems in adults with congenital heart disease and the second cause of death in these patients [1, 5].

In the second case, ECMO was used to treat a patient with cardiogenic shock after cardiac surgery. Although there is more experience in children [6], its use has also been described in adult patients after operations related to congenital heart disease [7]. Although ECMO is promising for stabilising postcardiotomy cardiogenic shock, survival is limited due to possible complications, of which haemorrhage is one of the most important. Prevention by withholding anticoagulants increases the risk of circuit clotting and thromboembolism [6]. Our first patient had a pre-existing coagulopathy, which made this consideration even more difficult.

In the second patient ECMO was started to treat multi-organ failure and as a bridge-to-decision. The use of ECMO in patients with multi-organ failure is not associated with a positive outcome, probably due to severe organ damage prior to the initiation of ECMO. Our patient suffered from acute renal failure at day one, while ECMO was started at day eight. Earlier initiation of ECMO may improve survival, as this can prevent organ damage [6].

As both cases illustrate, the use of ECMO is not without complications. Haemorrhagic problems, but also sepsis, mediastinitis and thrombosis, are frequent causes of death [6]. Inserting peripheral cannulas may damage vessels, making future interventions more difficult. Patients who have already undergone surgery have the disadvantage of scarring and changes in anatomy, which makes it more difficult to insert a central ECMO. Scarring due to the ECMO cannulas can precipitate new arrhythmias, which is the most frequent long-term complication and leading cause of death in this patient group [1, 5].

The use of extracorporeal membrane oxygenation may provide a valuable tool in stabilising patients with congenital heart disease who present with cardiogenic shock, especially when this is due to right ventricular failure [8]. The system can be used as a bridge-to-decision or as a bridge-to-surgery. Furthermore, it can be applied to overcome postcardiotomy cardiogenic shock.

Clinicians who apply ECMO should be aware of its serious risks and complications. If manifest multi-organ failure is already present, it is not recommended to start ECMO.
